# NRF2 signalling pathway: New insights and progress in the field of wound healing

**DOI:** 10.1111/jcmm.16597

**Published:** 2021-06-18

**Authors:** Yang Liu, Xiaofan Yang, Yutian Liu, Tao Jiang, Sen Ren, Jing Chen, Hewei Xiong, Meng Yuan, Wenqing Li, Hans‐Günther Machens, Zhenbing Chen

**Affiliations:** ^1^ Department of Hand Surgery Union Hospital Tongji Medical College Huazhong University of Science and Technology Wuhan China; ^2^ Department of Hand and Foot Surgery Huazhong University of Science and Technology Union ShenZhen Hospital Shenzhen China; ^3^ Department of Plastic and Hand Surgery Technical University of Munich Munich Germany

**Keywords:** NRF2, oxidative stress, ROS, wound healing

## Abstract

As one of the most common pathological processes in the clinic, wound healing has always been an important topic in medical research. Improving the wound healing environment, shortening the healing time and promoting fast and effective wound healing are hot and challenging issues in clinical practice. The nuclear factor‐erythroid–related factor 2 (NFE2L2 or NRF2) signalling pathway reduces oxidative damage and participates in the regulation of anti‐oxidative gene expression in the process of oxidative stress and thus improves the cell protection. Activation of the NRF2 signalling pathway increases the resistance of the cell to chemical carcinogens and inflammation. The signal transduction pathway regulates anti‐inflammatory and antioxidant effects by regulating calcium ions, mitochondrial oxidative stress, autophagy, ferroptosis, pyroptosis and apoptosis. In this article, the role of the NRF2 signalling pathway in wound healing and its research progress in recent years are reviewed. In short, the NRF2 signalling pathway has crucial clinical significance in wound healing and is worthy of further study.

**Abbreviations**
ALD: Alcoholic liver disease
ARE: Anti‐redox response elements
ASC: Apoptosis‐associated spot‐like protein
ATG: Autophagy‐related genes
caNRF2: Constitutively active NRF2
CAT: Catalase
CNC: Cap‘n'collar
DHM: Dihydromyricetin
ECM: Extracellular matrix proteins
ERK: Extracellular signal–regulated kinase 2
Fer‐1: Ferritin 1
GA: Gallic acid
GCLC: Glutamate‐cysteine ligase
GPx4: Glutathione peroxidase 4
HaCaT: Human immortalized keratinocytes
HFD: High‐fat diet
HMOX1(HO‐1): Haeme oxygenase 1
HO‐1: Haeme oxygenase 1
HUVEC: Human umbilical vein endothelial cells
KEAP 1: Kelch‐like ECH‐related protein 1
KO: Knockout
MnSOD: Mitochondrial local superoxide dismutase 2
mtROS: Mitochondrial ROS
NLRP3: NACHT, LRR and PYD domain‐containing protein 3
NQO‐1: NAD(P)H quinone dehydrogenase 1
NRF2 or NFE2L2: Nuclear factor‐erythroid–related factor
PA: Palmitic acid
PAI‐1: Plasminogen activator inhibitor 1
RIP: Receptor‐interacting protein
ROS: Reactive oxygen species
SIRT3: NAD‐dependent protein deacetylase sirtuin‐3
SLC7A11: Cystine/glutamate transporter x subunit Ç
sMAF: Small musculoaponeurotic fibrosarcoma protein
SOD: Superoxide dismutase
TfR1: Transferrin receptor

## 1 INTRODUCTION

### 1.1 Nuclear factor‐erythroid 2–related factor 2

Nrf2 belongs to the cnc (‘cap‘n'collar’) subfamily of the basic region‐leucine zipper transcription factors. So far, six members of this family have been identified: NF‐F2, Nrf1, Nrf2, Nrf3, Bach1 and Bach2. Nrf2 is considered to be the primary regulator in the process of oxidative stress.^1^ It exhibits seven conserved NRF2‐ECH homologous domains, and the function of Neh1 is to recognize the anti‐oxidative response element (ARE), which can interact with the small musculoaponeurotic fibrosarcoma proteins (sMAF) K, G and F, as well as bZip proteins, to form heterodimers and activate gene transcription. The kelch domain of kelch‐like ECH‐related protein 1 (KEAP1) specifically interacts and mediates the ubiquitination, and the degradation of NRF2 occurs at the Neh2 domain, which mainly contains ETGE and DLG motifs and is the core structure of the NRF2 area. The remaining domains, when combined with various components in the transcription mechanism, act as transcription activation domains, mediate the degradation of NRF2 in oxidatively stressed cells or bind to other receptors to regulate the activity of NRF2.^1^, ^2^, ^3^, ^4^, ^5^ The NRF2 regulatory pathway plays a vital role in protecting cells from oxidative damage. After exposure to electrophilic and oxidative stress, the reactive cysteine residues of Keap1 are modified, resulting in reduced E3 ligase activity, stable accumulation of NRF2 in cells and robust induction of a series of cytoprotective genes.^6^ During wound healing, free radicals, such as reactive oxygen species (ROS), are considered the main driving force of oxidative damage. The healing process of diabetic wounds is affected by the internal pathophysiology and external factors (repeated infection and trauma), such as reduced blood supply, matrix migration and wound contraction. Pretreatment with NRF2 activator reduced the oxidative stress level of diabetic wounds and promoted wound healing.^7^, ^8^ Given the high cost of wound treatment, it is essential to study this process regarding physical health and economic costs. Therefore, it is necessary and urgent to study the specific role of the NRF2 factor in wound healing.

### 1.2 Regulation of wound healing by NRF2

#### 1.2.1 The process of wound healing

**FIGURE 1:**
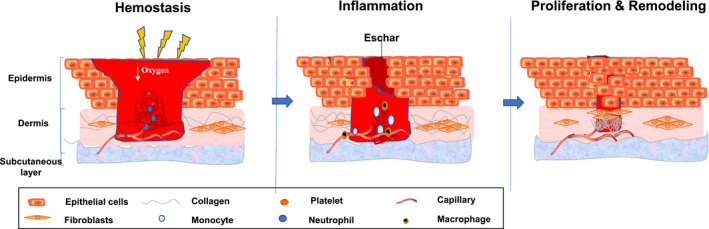
The process of wound healing. The process of wound healing includes four stages: haemostasis, inflammation, proliferation and remodelling. The cells involved in wound healing include macrophages, neutrophils, keratinocytes, monocytes, endothelial cells and fibroblasts

Wound healing and treatment are enduring topics in medical research, and although there have been numerous advances in understanding the steps involved in wound healing, the exact mechanisms underlying wound healing remain unclear. As one of the most complicated and miraculous physiological processes, wound repair occurs regularly in all living organisms^9^, ^10^ In current studies, the process of wound repair has been divided into four stages: haemostasis, inflammation, proliferation and remodelling (Figure 1).

Directly after tissue injury, the wound healing process begins. The wound starts to stop bleeding, and the damaged tissue cells release vasoactive substances to constrict the local blood vessels. At the same time, platelets aggregate and activate the coagulation system, and fibrinogen forms an insoluble fibrin network, produces blood clots, seals damaged blood vessels and protects the wound to prevent further bacterial contamination and loss of body fluids. Inflammation, a complex and massive body defence response with the purpose to remove or inactivate harmful substances, removes necrotic tissue and creates the right conditions for the subsequent proliferation process.^11^

The proliferative stage of wound healing is also called the granulation stage. It starts in the first week after the trauma and lasts about two to three weeks. New epidermal cells proliferate and divide to replace injured dead cells. The whole proliferative stage mainly includes three parts: cell proliferation and migration (re‐epithelialization), cell recruitment, and granulation tissue production. The epidermal cells at the edge of the wound migrate to the central area of the wound after temporary dedifferentiation. During the migration process, these cells rest in contact with the adjacent cells rather than immediately getting in contact with the wound edges. Therefore, epidermal cells continuously migrate and stagger in the wound and finally connect to form a complete epidermal layer. In detail, under the action of various growth factors, dermal fibroblasts can further differentiate into myofibroblasts and secrete large amounts of collagen and extracellular matrix proteins to the central area of the wound. It is well known that new granulation tissue is mainly composed of new capillary networks, fibroblasts, myofibroblasts and extracellular matrix protein (ECM), as well as inflammatory cells. A large number of fibroblasts and inflammatory cells often gather around capillaries. Moreover, inflammatory cells are usually dominated by macrophages and include many neutrophils and lymphocytes..^9^, ^12^

As the amounts of blood vessels and water in the granulation tissue decrease, the tissue in the wound hardens gradually to finally form scars. After wound repair, the hardened tissue softens and flattens, and the tissue intensity strengthens. Epithelial cells cover the wound due to proliferation and migration from the edge of the wound to its centre to form new epithelial cells. Decomposition and remodelling of the wound starts with the decomposition process, which comprises the three stages of wound healing and terminates with the end stage. The structures assembled in previous stages are removed or modified. As the edge of the migration layer aggregates, epidermal cell migration stops, and proliferation decreases. Extracellular matrix proteins are reshaped, and granulation tissue is eliminated. By dissolving the inside of the wound, a scar is formed by the aligned ECM filaments. Although failure or termination of any of the above processes can have catastrophic consequences due to bleeding or infection, a series of severe problems may also occur if any process takes longer than expected. For example, excessive or long‐term inflammation may slow down the process or even lead to aggravation of the original wound because the neutrophil collateral damage produced large amounts of ROS.^12^

In the process of normal wound healing, the level of reactive oxygen species is in a relatively balanced state. The redox signal mediated by the reactive oxygen species participates in different processes such cell recruitment and cytokine and growth factor production and plays a vital role in wound healing.^13^ When the body is in a state of disease, such as diabetes, this balance is broken, and the mitochondria produce considerable amounts of reactive oxygen species. By up‐regulating the polyol, hexosamine, and protein kinase C pathways and increasing the formation of glycation end products, endothelial cells are damaged, leading to local ischaemia and hypoxia.^14^ Simultaneously, oxidative stress injury results in reduced proliferation and migration of keratinocytes and fibroblasts, decreased synthesis of collagen and fibronectin, and imbalance of matrix metalloproteinases and their inhibitors, which further increases the wound healing difficulty.^15^ In addition, the sustained high ROS level causes chronic and long‐time inflammation of the wound, which further causes cell damage and delays wound healing.^13^ Therefore, oxidative stress injury is one of the critical factors of non‐healing wounds.

Therefore, wound repair is an extremely complex biological process in which transcription factors play a vital role.^16^, ^17^ NRF2 is one of the most representative transcription factors in the process of oxidative stress. Furthermore, the NRF2 signalling pathway takes part in the wound healing process. This article describes the role of the NRF2 signalling pathway in wound healing.

## 2 EXPRESSION AND ACTIVITY OF NRF2 IN WOUNDS

Oxygen (O_2_) is one of the substrates required for mitochondria to drive adenosine triphosphate (ATP) synthesis. In the process of wound repair, oxygen continuously supplies energy for tissue renewal and metabolism. At the same time, ROS, that is free radical derivatives of oxygen, are crucial. Members of the ROS family are molecules that usually contain O_2_ and are reduced to highly active free radical forms due to the electrons at the surface of these molecules. Typical members of the ROS family are the superoxide anion (⋅O_2_^−^), peroxide ion (⋅O_2_^−2^), hydrogen peroxide (H_2_O_2_), hydroxyl radical (OH) and hydroxyl ion (OH^−^). Under normal circumstances, the endogenous ROS source may be the endoplasmic reticulum or the oxidative phosphorylation of mitochondria during ATP production. The role of ROS in cell homeostasis shows that low ROS levels may inhibit cell growth and cause cell cycle arrest, while excessive ROS will induce the activation of pro‐apoptotic proteins and further induce cell death.^18^, ^19^

Therefore, many studies focused on the role of NRF2 in wound healing. Keratinocytes of the hyperproliferative epithelium in skin wounds showed a strong expression of NRF2, but NRF2 gene expression was also observed in granulation tissue cells.^20^ Further studies have shown that after full‐thickness injury in mice, the NRF2 gene increases with the increase of ROS and then gradually decreases with the healing of the wound. After activation, NRF2 can have the function of promoting epithelial repair. Further studies have shown that NRF2 activators or promoters are used as an effective means to promote cell healing. These reagents and factors include plasma,^21^, ^22^ bee venom,^23^ olive oil,^24^ curcumin,^25^, ^26^ neferine,^27^ emodin,^28^, ^29^ thymoquinone (Tq),^30^ resolvin D1 (RvD1),^31^ dermaprazole^32^ and various plant extracts.^33^, ^34^, ^35^, ^36^, ^37^, ^38^ Moreover, non‐coding RNA negatively regulates Keap1 upstream of NRF2 to indirectly promote the expression of NRF2.^39^ Umapathy *et al* showed that hyperbaric oxygen (HBO) therapy promotes wound healing by increasing the oxygen supply and distribution to damaged tissues, stimulating angiogenesis, reducing inflammation, increasing nitrate levels and promoting wound healing in the treatment of diabetic foot ulcers (DFU).^40^ Increased levels of NRF2 temporarily modulate the expression of vascular genes in wounds, which may accelerate the healing of chronic wounds. However, the mode of action of NRF2 is still uncertain, but NRF2 mainly targets the inflammation and proliferation phases of wound repair (Table 1).

**TABLE 1:** The reagents or factors target to nrf2 in wound repair

Category	Reagents/factors	Reagent properties	Cells/tissue	Models	Effects	Reference
The body composition	Cold plasma	\	Keratinocytes/dermal fibroblasts	Full‐thickness wound model in immunocompetent SKH1 mice	Promote the formation of granulation tissue and the stimulation of re‐epithelialization and proliferation, up‐regulation of vascular factors, and proliferation of new blood vessels	^22^
	MicroRNA‐24		Vascular smooth muscle cells (VSMCs)	STZ‐induced diabetic mice	Suppressed oxidative stress, promoted reendothelialization in balloon‐injured diabetic rats	^39^
	Adipose‐derived stem cell (ADSC) exosomes		Endothelial progenitor cells (EPCs)	STZ‐induced diabetic mice	Promoted proliferation and angiopoiesis	^43^
	Human embryonic stem cell exosomes (ESC‐Exos)		Endothelial cells	D‐galactose‐induced HUVEC senescence model.	Ameliorate endothelial senescence, recover ageing‐related angiogenic dysfunction, accelerating wound healing	^42^
Natural substances	Bee venom	\	Functional macrophages	STZ‐induced diabetic mice	Stimulating angiogenesis and correcting the impairment of diabetic wound healing	^23^
	Curcumin	Stilbene	Primary rat astrocyte	MeHg‐induced cell toxicity	Neuroprotection	^25^
		Stilbene	Adult skin fibroblasts	Lifespan completed (LSC) theory	Cell proliferation and migration	^26^
	Neferine	Bisbenzylisoquinoline alkaloid	Skins/macrophages	STZ‐induced diabetic mice	Improved wound contraction, epithelialization and modulation of inflammatory	^27^
	Emodin	Anthraquinone compound	Madin‐Darby canine kidney (MDCK) cells and A549 lung cancer cells	Influenza A virus (IAV) infection model	Increase the survival rate, reduces lung oedema, viral titter and inflammatory cytokines, and improves IAV‐induced histopathological changes	^28^
		Anthraquinone compound	Human neuronal SH‐SY5Y cells	OGD/reoxygenation (OGD/R)	Emodin protected cells from OGD/R‐induced apoptosis.	^29^
	Thymoquinone(Tq)	Thymoquinone(Tq)	Kidney tissues	Male Sprague Dawley rats	Tq treatments up‐regulated HO‐1 via activation of Nrf2 to activate antioxidant enzymes.	^30^
	Ginsenoside Rg1	Saponin	Interleukin‐1β (IL‐1β) treated podocytes	Anti‐GBM GN mouse model	Ginsenoside Rg1 attenuates IL‐1β‐induced inflammation and apoptosis in podocytes	^33^
	Ginsenoside Re	Saponin	Human neuronal SH‐SY5Y cells	Amyloid‐β (Aβ) inducted AD model	Strong neuroprotective activity antioxidant	^34^
	Plumbagin	Alkaloid	Mouse skins	STZ‐induced diabetic mice	plumbagin minimized the oxidative stress and improved antioxidant status	^36^
	Caffeic acid phenethyl	Phenolic compound	Macrophages	Pressure ulcers	Promoting re‐epithelialization, dermal reconstruction and wound closure	^37^
	Dimethyl fumarate	Fumaric acid ester	keratinocytes	STZ‐induced diabetic mice	Attenuated oxidative damage and inflammation, accelerated wound closure in diabetic mice	^38^
	Resolvin D1 (RvD1)	Polyunsaturated fatty acid	The corneal epithelium	STZ‐induced diabetic mice	Promote the regeneration of corneal epithelium, increase the synthesis of GSH, reactivate the NRF2/ARE signal pathway and promote the regeneration of corneal nerves	^31^
Chemical compounds	Dermaprazole	Proton pump inhibitors (PPIs)	3D human skin	Mouse model of radiation‐induced dermatitis	The early induction of endogenous antioxidant defence mechanisms and down‐regulation of pro‐inflammatory and pro‐fibrotic mechanisms	^32^
	RTA 408	Synthetic triterpenoids	Skins	Leprdb/db mice	Systemic or simple topical application of the Nrf2‐inducing semi‐synthetic oleanane triterpenoid, RTA 408, significantly accelerates diabetic wound healing	^35^

It has recently been demonstrated that exosomes, as natural nanoparticles secreted from cells, play a crucial role in cell‐to‐cell communication.^38^, ^41^, ^42^ Chen *et al* showed that ESC exosomes improve endothelial cell ageing by activating NRF2 and restore ageing‐related angiogenesis dysfunction, thereby accelerating wound healing in ageing mice.^8^ Moreover, Li *et al* believed that exosomes can reduce inflammation by preventing EPC ageing and inhibiting the expression of ROS and inflammatory cytokines to support wound healing by improving angiogenesis, thereby reducing the progression of DFU in diabetic patients.^43^, ^44^ However, Li *et al* reported that the down‐regulation of miR‐200a in ESC exosomes partially eliminated the effects of ESC exosomes on Nrf2 activation and HUVEC ageing, indicating that other mechanisms may be involved in these processes. Chen *et al* showed that although ADSC‐derived exosomes can potentially promote wound healing in diabetic mice, no clinical report proved to date whether it can promote the healing of diabetic foot ulcers. Therefore, Nrf2 exosome therapy is an exciting new research direction and whether it plays a role in the healing process of human wounds remains to be verified.

## 3 EFFECT OF NRF2 DEFICIENCY ON WOUND HEALING

The effect of NRF2 activation on wound healing has been described in detail above, and the lack of NRF2 in wound healing has also been studied in depth.

Current research on pharmacological inhibitors of Nrf2 mainly focuses on the direction of cancer. However, there is still controversy about the role of Nrf2 in cancer. Studies have shown that Nrf2 knockout (KO) mice are sensitive to chemically induced cancer changes, suggesting that Nrf2 can limit cancer formation to a certain degree.^45^, ^46^ On the other hand, Nrf2 is overexpressed in many different types of tumours, and it is associated with poor prognosis of cancer (including promoting the proliferation of cancer cells and increasing the resistance of cancer cells to chemotherapeutic drugs).^47^, ^48^ Therefore, we can cautiously assume that Nrf2 inhibitors can make anti‐cancer therapies more effective and sensitive. However, since the inhibitory mechanisms are unknown or not specific, it will take a long time for Nrf2 inhibitors to move from laboratory to clinical use. Regarding the application of Nrf2 inhibitors in the wound healing process, Chen *et al* treated the NRF2 inhibitor brusatol with Esc‐Exos and reported that brusatol treatment eliminated the down‐regulation of ROS activity mediated by ESC‐Exos.^42^

Braun *et al* observed that the collagen production at and around the wounds of Nrf2 KO mice and the excessive expression of pro‐inflammatory cytokines by macrophages caused delayed wound healing. Despite the observed differences in cytokines, Nrf2 KO and control mice showed no differences in the wound healing dynamics, which may be at least partly due to a compensatory effect of other transcription factors (such as Nrf1 or Nrf3).^20^

The dominant‐negative Nrf2 mutant dnNrf2 expressed in the epidermis of transgenic mice lacks the binding domain and transactivation domain of KEAP1. In general, dNrf2 mutants can aggregate in the nucleus and bind to ARES (antioxidant response element) without promoting transcription. This feature can prevent other transcription factors (such as cap‘n'collar transcription factors) from binding to AREs, thereby effectively avoiding the compensation caused by NRF2 deletion. However, one study found that keratinocyte‐specific dnNRF2 mutant mice did not show significant differences in normal skin and wound healing processes.^49^ NRF2 may have different functions in different cells and different environments, face different degrees of environmental stress and have different target genes.^50^, ^51^ Hiebert *et al* showed in vitro that skin fibroblasts lacking NRF2 exhibited a slight increase in the ROS level, while no differences in their proliferation and wound healing kinetics were observed compared with the control group.^52^ Therefore, it needs to be emphasized that NRF2 plays a specific role in wound healing. NRF2‐deficient mice had prolonged wound inflammation time, suggesting that NRF2 deficiency may have a certain effect on the recruitment of immune cells.^20^ The immune cells of the myeloid lineage have essential functions in wound healing, as they can resist invading bacteria and produce factors that promote healing by producing ROS.^11^ Although only small amounts were detected in the wounds of wild‐type mice, NRF2 and its cytoprotective target gene NQO‐1 are strongly expressed in macrophages and neutrophils. Myeloid‐specific Nrf2 KO mice are sliced and immunized by histological wounds, and cell quantitative analysis showed no noticeable difference in wound healing.^53^

Most recent wound healing studies based on NRF2 have focused on the detection of wound closure. Although NRF2 may play an important role, it may not be essential for wound healing in healthy mice that do not face additional challenges. Only few studies have been reported on the influence of scar formation in the later stage of wound healing. Future research may answer these unsolved problems.

## 4 NRF2, A POTENTIAL THERAPEUTIC STRATEGY FOR WOUND REPAIR

In general, NRF2 activators continue to attract significant attention as therapeutic agents due to their antioxidant properties under pathological conditions. The functional role of NRF2 in wound repair has always been the focus of clinical research. In recent years, exosomes, membrane lipid vesicles with a diameter of about 30‐100 nanometres, have received more and more attention due to their role in communication between cells.^54^ Li *et al* showed that adipose‐derived mesenchymal stem cell exosomes can prevent EPC ageing, mainly when NRF2 is overexpressed. They also control inflammation by inhibiting the expression of ROS and inflammatory cytokines, thereby further improving angiogenesis and promoting wound healing,^43^ which provides the possibility for the clinical application of NRF2. Compared with the traditional electrophilic NRF2 activator, directly inhibiting the interaction between Keap1 and NRF2 has more advantages. Development and utilization of Keap1‐NRF2 protein‐protein interaction inhibitors have become emerging research topics, and potential inhibitors of this target have been identified.^55^ Treatment of the wounds of diabetic mice with KEAP1 siRNA reduces delayed wound healing and increases the formation of granulation tissue.^56^, ^57^ However, studies have shown that irregular keratinization and hyperkeratosis occur in the oesophagus of KEAP1 KO mice, causing stomach obstruction. Therefore, the survival period of KEAP1 KO mice does not exceed two to three weeks.^58^ Due to the lethality of KEAP1 KO mice, constructing cell‐specific expression of constitutively active NRF2 (caNRF2) mutants is an effective alternative. Due to the lack of the KEAP1 binding domain to achieve the specific expression of NRF2, which does not affect the interaction between KEAP1 and other proteins, the caNRF2 mutant still has constitutive activity in the nucleus.^59^, ^60^

As one of the primary cells involved in the wound healing process, activated Nrf2 fibroblasts play an indispensable role in the whole wound healing process. Relevant studies have shown that the activation of NRF2 in fibroblasts accelerates the senescence of fibroblasts in vivo or in vitro. The main reason is that, mediated by NRF2, the senescence‐promoting factor plasminogen activator inhibitor 1 (PAI‐1, serpine1) in the matrix accelerates the senescence of cells, although it reduces the ROS level and DNA damage. However, in mice expressing caNRF2 fibroblasts, the wound healing time is shortened, and senescent cells can directly promote the proliferation of keratinocytes.^52^ The activation of NRF2 in inflammatory cells seems to have a relatively small effect on wound healing. Mice expressing caNRF2 in bone marrow cells showed no significant difference in the wound closure rate and even failed to show a significant increase in the expression of NRF2 target genes.^53^ A possible reason is that, even under steady‐state conditions, the NRF2 expression level in these immune cells (such as neutrophils) is still high, so the specific expression of caNRF2 is not evident or does not occur. Combined with the above studies, in the case of adverse effects on wound healing (such as oxidative stress and increased ROS levels), activating the NRF2/HO‐1 pathway is a potential treatment strategy to promote wound repair.

## 5 REGULATION OF NRF2 PATHWAY ACTIVATION

### 5.1 Regulation of calcium

In homeostasis, due to the Na^+^‐K^+^‐ATP and Ca^2+^ pumps on the bimolecular phospholipid membrane, the charged ions are in a relatively balanced state inside and outside the cell membrane. In addition to Na^+^ and K^+^, the current changes inside and outside the cell also play an indispensable role. The excessive influx of Ca^2+^ into cells may lead to excessive amounts of mitochondrial Ca^2+^, which could induce apoptosis and activate Ca^2+^‐dependent degradation enzymes, leading to oxidative stress and cell dysfunction. Jin *et al* confirmed that in the cholesterol‐mediated oxidative damage of vascular endothelial cells, the activation of Nrf2 and MAPK/ERK signalling pathways and increase of the Ca^2+^ concentration could increase HO‐1 expression. In contrast, Nrf2 expression increases with the increase of the Ca^2+^ concentration, and activation of the ERK signalling pathway causes HO‐1 overexpression. Overexpression of HO‐1 can inhibit the transduction of the PI3K/Akt signalling pathway and affect the expression of downstream c‐myc to reduce cell damage.^61^ Many studies have shown that, as a classical anti‐oxidative stress signal transduction pathway, the NRF2 signalling pathway is the focus of research with great potential to provide strategies for repairing blood vessels and nerves during wound injury.

### 5.2 Relationship between mitochondrial oxidative stress and NRF2 pathway

Among the various sources of reactive oxygen species (ROS) in cells, electrons released by the mitochondrial electron transport chain present the most important ROS source. In mitochondria, molecular oxygen is reduced to the superoxide anion (O2^•‐^), which is then converted into H_2_O_2_ to produce more active hydroxyl groups (•OH). Under the action of catalase or glutathione peroxidase, hydrogen peroxide is inactivated. Among the external factors that cause oxidative stress, there are two main reasons for mitochondrial dysfunction: One is the mitochondrial electron transport dysfunction and the other is protein degradation caused by the abnormal accumulation of ROS. The opening of the mitochondrial membrane channel further damages the mitochondrial membrane structure. The resulting mitochondrial degradation leads to a lack of ATP. In combination with the incomplete mitochondrial membrane, intracellular Ca^2+^ overload and insufficient autophagy, wound healing is delayed or does not take place.^62^

Detailed analysis of the relationship between ageing functions can provide new strategies for improving the efficacy of stem cell therapy. Abnormal homeostasis of mitochondrial ROS (mtROS) is an important factor that causes cell senescence. Studies have shown that deacetylase SIRT3 regulates mtROS activity, and the expression level is controlled by mitochondrial local superoxide dismutase 2 (MnSOD). SIRT3 expression is mainly regulated by NRF2, so targeted NRF2 therapies may enhance the therapeutic effect of mesenchymal stem cells on skin wound healing.^63^ Therefore, treatment strategies for mitochondrial ROS and oxidative stress may be potential treatment strategies for wound healing.

### 5.3 NRF2 signalling pathway in ferroptosis

Ferroptosis, a new type of non‐apoptotic cell death that is characterized by lipid peroxidation, is primarily dependent on iron and reactive oxygen species (ROS). The iron‐promoting effect is mainly triggered by the activation of antioxidants in the cell, leading to the accumulation of lipid ROS and destruction of the membrane structure.^64^ For ferroptosis, the dynamic balance between oxidative stress and antioxidants determines the lipid peroxidation level in cells. This new type of cell death is thus closely related to the lipid peroxidation of cells.

NRF2 acts as a core transcription factor in the pathway of oxidative stress and has some target genes, such as glutamate‐cysteine ligase (GCLC), cystine/glutamate transporter x subunit Ç (SLC7A11), NAD(P)H quinone dehydrogenase 1 (NQO‐1) and haeme oxygenase 1 (HO‐1). Among these target genes, GCLC and SLC7A11 are involved in GSH synthesis, lipid peroxidation, ROS and ferroptosis, as well as the synthesis of the inhibitor GPx4.^65^, ^66^ Furthermore, these target genes are also involved in iron metabolism proteins, such as transferrin receptor (TfR1), iron transporter (Fpn) and ferritin‐1 (Fer‐1), as well as the regulation of haeme oxygenase 1 (HO‐1).

Michele *et al* showed that Gclc‐deficient mice exhibited epidermal hyperkeratosis, reduced adhesiveness of the corneocytes and glutathione‐deficient keratinocytes. Additional loss of Nrf2 did not aggravate the phenotype, demonstrating that the cytoprotective effect of Nrf2 is glutathione‐dependent.^64^ Thus, Michele *et al* hypothesized that the cell‐protective effect of NRF2 is strongly induced by enzymes involved in glutathione synthesis and recovery. In the absence of Gclc, this protective effect is no longer present, so the regulation of Nrf2 in Gclc‐deficient cells does not result in a functional rescue. Therefore, in addition to the regulatory relationship between Gclc and Nrf2, the crosstalk between NRF2 and ferroptosis still needs further research and discovery in the field of wound healing.

### 5.4 NRF2 signalling pathway in pyroptosis

Pyroptosis, which is characterized by the release of inflammasomes, the formation of ASC (apoptosis‐associated spot‐like protein) and the activation of pro‐inflammatory caspase, is a widely recognized form of programmed cell death.^67^, ^68^ The NRF2 signalling pathway is closely related to apoptosis during inflammation. In the renal ischaemia‐reperfusion model, PRMT (protein arginine methyltransferase) can be inhibited by activating the NRF2/HO‐1 pathway, thereby regulating oxidative stress and apoptosis and promoting the proliferation of renal tubular epithelial cells. Many different stress factors activate NLRP3 as an inflammasome, and the NRF2 signalling pathway regulates redox homeostasis and the protective response of cells.^69^ Hu *et al* showed that in HUVEC (human umbilical vein endothelial cells), DHM (dihydromyricetin) can inhibit the PA‐induced activation of ROS‐dependent NLRP3 (NACHT, LRR and PYD domain‐containing protein 3) inflammasomes by activating the NRF2 signalling pathway to improve cell apoptosis and reduce secreted IL‐1b.^70^

### 5.5 NRF2 signalling pathway in autophagy

Autophagy is a biological process by which eukaryotic cells maintain cell homeostasis and perform self‐renewal. This process has become another important research field after apoptosis. Autophagy can be divided into three types: macroautophagy, microautophagy and chaperon‐mediated autophagy, but currently, it usually refers to the research of macroautophagy. As a dynamic biological process, autophagy includes several stages: initiation, expansion and maturation. Autophagy‐related (ATG) genes are regulators in this process that take part in every stage of the process and strictly regulate each stage.^71^ The induction of autophagy promotes migration under certain conditions but is related to the inhibition of cell migration under other conditions.

Studies have shown that long‐term exposure to arsenite can reduce autophagy by blocking the induction and inhibition of autophagy degradation, and the changes in autophagy are related to the overexpression of NRF2 in HaCaT cells. Arsenite promotes the up‐regulation of NRF2 and downstream mTOR through the PI3K/Akt pathway, followed by the inhibition of autophagy initiation.^72^, ^73^

Studies have pointed out that the up‐regulation of NRF2‐dependent gene expression in keratinocytes may be related to the genetic inhibition of autophagy. Inoue *et al* confirmed that the excessive inhibition of autophagy in the liver and lung epithelial cells leads to the activation of the NRF2 pathway, which in turn up‐regulates the downstream target genes of NRF2, causing severe tissue damage.^74^, ^75^ Furthermore, Jain *et al* suggested that in Atg7 knockout keratinocytes, the p62‐NRF2 feedback loop enhances the NRF2 activity.^76^ As a possible reason, they hypothesized that the degradation of KEAP1 is reduced, and oxidized phosphatidylcholines directly affect NRF2.^6^ In this case, studies on genetically modified mice have shown that NRF2 is a double‐edged sword, and persistently high levels of NRF2 may not have a protective effect. NRF2 may also interfere with the homeostasis of the cell epidermis.^77^ In conclusion, autophagy is a relevant mechanism for limiting the NRF2 activity in the dermis.

### 5.6 NRF2 signalling axis in programmed cell necrosis

Programmed cell necrosis is a new type of cell death, which usually has similar morphological characteristics to cell necrosis but is regulated by a unique death signal pathway. Receptor‐interacting proteins (RIP) 1 and 3 are vital regulatory proteins in the nec signalling pathway. RIP1 is the intersection of cell survival and death, and RIP3 is the converter that determines the mode of cell death (ie apoptosis or nec).

Ge *et al* showed that the activation of NRF2 can effectively inhibit the production of ROS and the expression of RIP3 in PAL‐stimulated cells, as well as inhibit cell inflammation and lipid accumulation, thereby regulating hepatic steatosis induced by high‐fat diet (HFD).^78^ Similarly, the main factors that cause alcoholic liver disease (ALD) damage are liver cell inflammation and excessive death. Zhou *et al* showed that gallic acid (GA) can reduce the expression of necrotic RIP1 and RIP3 and release high‐mobility basic box protein 1 to promote NRF2 to inhibit hepatocyte necrosis.^79^ The very close relationship between the NRF2 pathway and procedural necrosis presents a potential research direction in the field of wound repair.

### 5.7 NRF2 signalling pathway in oxeiptosis

In 2018, the term oxeiptosis was proposed by Andreas Pichlmair's laboratory. Oxeiptosis is a new type of regulated cell death that is characterized by the activation of oxygen free radicals by the KEAP1‐PGAM5‐AIFM1 pathway and that is independent of caspase. Pichlmair *et al* reported the reaction of mice to ozone and the effect of hydrogen peroxide (H_2_O_2_) to epithelial cells and fibroblasts.^80^ It is known that the KEAP1‐NRF2 ‐HO‐1 pathway mediates the protective effect of cells against oxidative stress damage, but over‐activated KEAP1 can mediate H_2_O_2_‐induced oxidative steatosis independently of NFE2L2. Ozone‐ or H_2_O_2_‐induced oxeiptosis is independent of apoptotic or pyroptotic caspases, necroptosis, autophagy and ferroptosis. Ozone‐ or H_2_O_2_‐induced oxidative epidermopathy is distinguished from apoptosis or pyrolysis, autophagy, necrosis and ferroptosis.

### 5.8 NRF2‐regulated antioxidants involved in the antioxidant mechanism of cells in the NRF2 pathway

**FIGURE 2:**
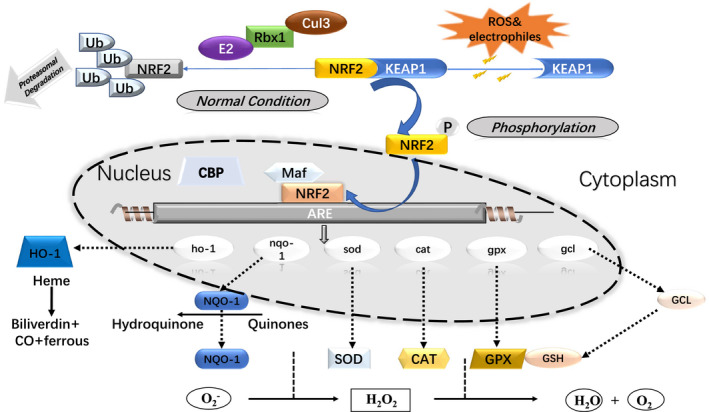
Schematic model of the Nrf2‐Keap1 signalling pathway. Under normal condition, Keap1 binds to Nrf2 and brings Nrf2 into Keap1‐Cul3‐E3 ubiquitin ligase complex, leading to ubiquitination and subsequent degradation of Nrf2. When Nrf2 met oxidative stress or electrophiles, it can cause a conformational change in the Keap1‐Cul3‐E3 ubiquitin ligase by acting on specific cysteine residues in Keap1. Also, the loss of cullin 3 could lead to Nrf2 activation. These changes disrupt Nrf2‐Keap1 binding at the DLG domain. Nrf2 is stabilized, and free Nrf2 translocates to the nucleus, where it dimerizes with members of the small Maf family and binds to AREs within regulatory regions of a wide variety of cell anti‐oxidative genes, including HO‐1, NQO‐1, SOD, CAT, GPX and GCL

NRF2‐regulated antioxidants involved in the antioxidant mechanism of cells in the NRF2 pathway mainly include antioxidants of cells and their related proteins, as well as antioxidant enzymes. These antioxidants mainly act in two ways: One is the role of ROS (CAT and SODs) and the other is the interaction with reductants (GPX, glutathione, *etc*), which is described in the following text (Figure 2).

#### 5.8.1 GCL

Glutathione can be used as a cell reducing agent due to its active thiol (‐SH) group. Glutamic cysteine ligase (GCL) and glutathione synthase (GS) catalyse the multi‐regulated two‐step process of glutamic cysteine ligase synthesis. γ‐GCL is a heterodimer composed of GCL catalyst (GCLC) and GCL modifier (GCLM). It enhances the catalytic reaction by decreasing the Km of glutamate in the substrate during the oxidation reaction. NRF2 is the upstream regulator of GCLC and GCLM.^81^, ^82^, ^83^

#### 5.8.2 GPX

GPX is a strong reductant. In its oxidized state, it simultaneously releases electrons by GSSH and mainly catalyses the formation of peroxide or organic hydrogen peroxide.^84^, ^85^ The activity of GPX is regulated by the NRF2‐ARE axis.

#### 5.8.3 SODs and CAT

Studies have shown that both SODs and CAT are downstream target genes of NRF2. SOD is the oxygen free radical with the highest oxidation potential among ROS molecules. Therefore, a two‐step process is required for the elimination of SOD, and SODs and CAT are the main regulators of these two reactions. First, SODs are decomposed into hydrogen peroxide and water, and then CAT further decomposes the formed hydrogen peroxide into water and oxygen, completely scavenging the oxygen free radicals.^18^, ^86^, ^87^ Different types of SOD with different subcellular location and type of cofactor required are known: Copper‐zinc superoxide dismutase (Cu/ZnSOD) was found in the mitochondrial cytoplasm, while manganese superoxide dismutase (MnSOD) was found in the mitochondrial matrix.

##### HMOX‐1 (HO‐1)

As a crucial catalytic decomposition product of haeme metabolic enzyme, HO‐1 is involved in b‐type oxidative cracking and produces carbon monoxide haemoglobin, biliverdin and Fe^2+^. Furthermore, biliverdin reductase catalyses the reduction of biliverdin to bilirubin, which is resistant to ROS active oxygen and reduces cell damage caused by ROS.^88^, ^89^

##### NQO‐1

In cells, quinone compounds are usually converted into relatively unstable semi‐quinone compounds by cytochrome P450 and NADPH, which can react with the sample to generate reactive oxygen damage to cells. The main function of NQO‐1 is to prevent cellular oxidative damage induced by quinone. NQO‐1 competes with NADPH and cytochrome P450 to maintain intracellular redox homeostasis by converting quinones into relatively stable hydroquinones through a two‐electron reduction process wherein NADH or NADPH acts as a reduction cofactor.^90^ However, studies have found that the regulatory role of NRF2 leads to methylation of the promotor region of NQO‐1 to activate its transcription. Therefore, NQO‐1 is also regulated by NRF2 transcription.^88^

## 6 SUMMARY AND FUTURE PERSPECTIVES

**FIGURE 3:**
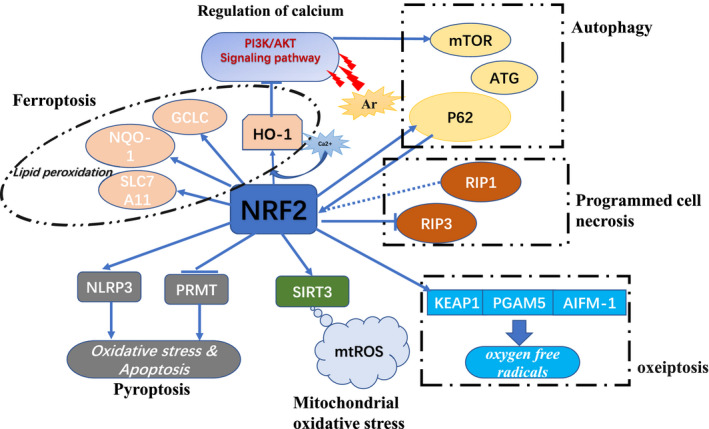
The main regulation modes of NRF2 signalling pathway. The regulation of NRF2 signalling pathway mainly includes the following: regulation of calcium ion (the overexpression of HO‐1 is caused by the increase of calcium ion concentration, and the overexpression of HO‐1 can inhibit the transduction of PI3K/AKT signalling pathway), mitochondria oxidative stress (NRF2 is the main transcription factor expressed by SIRT3, so the expression of SIRT3 can be enhanced by regulating NRF2 to optimize the therapeutic effect of mesenchymal stem cells on skin wound healing), ferroptosis (NRF2 downstream target genes HO‐1, GCLC, NQO‐1 and SLC7A11, by participating in lipid peroxidation), pyroptosis (activate the Nrf2 pathway, promote cell apoptosis and inhibit the activation of inflammasome NLRP3), autophagy (arsenite affects the expression of Nrf2 and mTOR through the PI3K/AKT pathway, as well as its effect on P62 and ATG genes), programmed cell necrosis (the regulatory relationship between Nrf2 and receptor‐interacting proteins RIP1 and RIP3) and oxeiptosis (activates oxygen free radicals through the KEAP1‐PGAM5‐AIFM1 pathway, independent of caspase)

As the most prominent molecule of the oxidative stress pathway, NRF2 and its anti‐oxidative stress effect have been the focus of an immense number of studies. In addition to its role in wound healing, NRF2 also plays a critical role in preventing cancer and treating chronic diseases.^91^, ^92^ However, it is worth noting that, in addition to its cytoprotective effect, NRF2 promotes the senescence of fibroblasts in the skin, the proliferation of keratinocyte stem cells, the proliferation of sebaceous glands and the abnormal deposition of ECM in STZ‐induced diabetic mice.^52^, ^93^ Keratinocytes, as the primary cell type involved in the wound healing process, can promote their own proliferation and migration and inhibit cell apoptosis when NRF2 is activated.^8^ As a multi‐organ protection pathway, the NRF2 signalling pathway has anti‐oxidative stress and anti‐apoptosis effects, promotes the re‐epithelialization and mediates matrix remodelling. The NRF2 signalling pathway has become a potential research direction for studying the process of wound healing. Complex regulatory mechanisms occur in oxidative stress–related wound healing, such as the reduction of mitochondrial damage, regulation of calcium ions in the cell, programmed cell death, autophagy and ferroptosis (Figure 3). Activation and suppression of the NRF2 signalling pathway have a vital impact on wound healing. The in‐depth study of the NRF2 signal transduction pathway can provide reliable theoretical support for clinical research on wound healing. Therefore, it needs to be clarified if NRF2 plays a crucial role in the human wound.

## CONFLICT OF INTEREST

The authors declare that they have no competing interests.

## AUTHOR CONTRIBUTIONS

**Yang Liu:** Conceptualization (equal); Writing‐original draft (lead). **Xiaofan Yang:** Visualization (equal). **Yutian Liu:** Formal analysis (equal); Visualization (equal). **Sen Ren:** Investigation (equal); Supervision (equal). **Jing Chen:** Data curation (equal); Supervision (equal). **Tao Jiang:** Methodology (equal); Validation (equal). **Hewei Xiong:** Data curation (supporting); Validation (supporting). **Meng Yuan:** Methodology (equal); Validation (supporting). **Wenqing Li:** Methodology (supporting); Resources (supporting). **Hans‐Guenther Machens:** Investigation (equal); Visualization (supporting). **Zhenbing Chen:** Funding acquisition (equal); Writing‐review & editing (equal).
